# Polo-like kinase and Aurora B kinase phosphorylate and cooperate with the CIF1-CIF2 complex to promote cytokinesis initiation in *Trypanosoma brucei*

**DOI:** 10.1098/rsob.220197

**Published:** 2022-10-05

**Authors:** Yasuhiro Kurasawa, Kyu Joon Lee, Huiqing Hu, Kieu T. M. Pham, Ziyin Li

**Affiliations:** Department of Microbiology and Molecular Genetics, University of Texas Health Science Center at Houston, Houston, TX 77030, USA

**Keywords:** *Trypanosoma brucei*, cytokinesis, Polo-like kinase, Aurora B kinase, CIF1, CIF2

## Abstract

Cytokinesis in eukaryotes is regulated by a Polo-like kinase-mediated and Aurora B kinase-mediated signalling pathway that promotes the assembly of the actomyosin contractile ring, a cytokinesis machinery conserved across evolution from yeast to humans. *Trypanosoma brucei*, an early divergent parasitic protozoan, employs an actomyosin-independent mechanism for its unusual cytokinesis that is controlled by a regulatory pathway comprising the Polo-like kinase TbPLK, the Aurora B kinase TbAUK1 and multiple trypanosomatid-specific regulators. However, whether any of these trypanosomatid-specific regulators function as substrates of TbPLK and/or TbAUK1 and how they cooperate with TbPLK and TbAUK1 to promote cytokinesis remain unknown. Here, we demonstrate that TbPLK and TbAUK1 phosphorylate the cytokinesis regulators CIF1 and CIF2 on multiple sites within their intrinsically disordered regions. We further show that TbPLK localization depends on its interaction with CIF1 from S/G2 phases, that TbPLK maintains CIF1 and CIF2 localization from G2 phase until early mitosis, and that TbAUK1 maintains CIF1 and CIF2 localization from late mitosis. Finally, we demonstrate that the cytokinesis regulators CIF4 and FPRC are not substrates of TbPLK and TbAUK1, and that they function upstream of TbPLK and TbAUK1 in the cytokinesis regulatory pathway. Together, these results provide insights into the functional interplay and the order of actions between the two protein kinases and the trypanosomatid-specific cytokinesis regulators in *T. brucei*.

## Graphical abstract

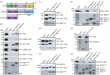

## 1. Introduction

Cytokinesis is the final step of the cell division cycle in all living organisms and is a tightly controlled process that requires the coordinated actions of numerous regulatory proteins at the site of cytokinesis initiation and along the cleavage furrow [1]. Organisms from different domains of life are known to use distinct molecular machineries for cytokinesis, with bacteria and some archaea species employing the FtsZ contractile ring and most of the eukaryotes, such as amoeba, fungi and animals, employing the actomyosin contractile ring [2]. The actomyosin-dependent cytokinesis regulatory pathway in fungi and animals involves two highly conserved protein kinases, the Polo-like kinase and the Aurora B kinase, which cooperate to regulate certain downstream factors to activate the formation of the actomyosin contractile ring [3]. These two protein kinases phosphorylate a central spindle-localized protein complex named the centralspindlin, a heterotetrameric protein complex consisting of two copies each of the kinesin protein MKLP1 (mitotic kinesin-like protein 1) and the Rho GTPase activating protein (RhoGAP) named MgcRacGAP [1]. The centralspindlin complex plays multiple roles by functioning as a kinesin motor with microtubule bundling activity, a scaffold for recruiting certain cytokinesis regulators, a conventional RhoGAP, and an anchor for linking the central spindle to the plasma membrane [4]. At the central spindle, the centralspindlin complex recruits Ect2, a guanine nucleotide exchange factor, to the midbody/cleavage furrow, where Ect2 activates the small GTPase RhoA for the latter to promote the assembly of the actomyosin contractile ring complex [1]. Despite the existence of certain unique cytokinesis regulators and/or pathways in different eukaryotic organisms, the actomyosin-based cytokinesis regulatory pathway is well conserved across evolution from yeast to humans.

Actomyosin-independent mechanisms for cytokinesis exist in many early divergent protozoans that lack the homologue of myosin II, a key component of the actomyosin contractile ring [5]. One of those protists that lack the actomyosin-based cytokinesis machinery is *Trypanosoma brucei* [6], a protozoan parasite causing human sleeping sickness in sub-Saharan Africa. A *T. brucei* cell possesses a highly polarized microtubule cytoskeleton and a motile flagellum that is adhered, for most of its length, to the cell membrane via the flagellum attachment zone (FAZ), which is located at the junction between the flagellum and the cell membrane [7]. During the cell division cycle, a trypanosome cell duplicates and segregates its flagellum and FAZ, and the distal tip of the newly assembled intracellular FAZ filament extends to the anterior tip of the new-flagellum daughter cell, which constitutes the cytokinesis initiation site [8]. Cleavage furrow ingression starts from the anterior tip of the new-flagellum daughter cell and proceeds uni-directionally, along the pre-formed cell division fold, towards the nascent posterior of the old-flagellum daughter cell [9,10]. Finally, the thin thread of cytoplasm connecting the nascent posterior of the old-flagellum daughter cell to the new-flagellum daughter cell is cleaved by yet unknown mechanisms, thereby completing cytokinesis to generate two daughter cells.

Regulation of the unusual cytokinesis in *T. brucei* requires the Polo-like kinase homologue TbPLK [11,12] and the Aurora B kinase homologue TbAUK1 [13,14], which act sequentially at the distal tip of the new FAZ or the anterior tip of the new-flagellum daughter cell to promote cytokinesis initiation [15,16]. The cytokinesis regulatory proteins that function downstream of TbPLK and upstream of TbAUK1 in the cytokinesis signalling pathway include a subset of trypanosomatid-specific proteins, such as CIF1 (also known as TOEFAZ1) [16–18], CIF2 [19] and CIF3 [20]. Inhibition of the kinase activity of TbPLK and TbAUK1 with small-molecule inhibitors and knockdown of TbPLK and TbAUK1 by RNAi reduce the levels of phosphorylated forms of CIF1 [16], identifying CIF1 as a potential substrate of both protein kinases. Additional cytokinesis regulatory proteins that function in the CIF1-mediated pathway at the new FAZ tip and/or the cleavage furrow include the kinetoplastid-specific protein phosphatase KPP1 [21–24], the trypanosomatid-specific proteins CIF4 and FPRC [25], the orphan kinesin KLIF [22,23], and the microtubule-severing enzyme complex KAT80-KAT60a [22]. KLIF and the KAT80-KAT60a complex are required for furrow ingression and cytokinesis completion [22,23,26], and the other cytokinesis regulators, CIF1-CIF4, FPRC and KPP1, are required for cytokinesis initiation [16,18–23,25]. Some of the cytokinesis regulators, including TbAUK1, CIF1, CIF3, CIF4 and FPRC, additionally localize to the cleavage furrow during cytokinesis; therefore, they may play additional roles in furrow ingression and cytokinesis completion.

In this report, we investigated the candidacy of trypanosomatid-specific cytokinesis regulators, CIF1, CIF2, CIF4 and FPRC, as substrates of TbPLK and TbAUK1 by identifying the *in vitro* phosphosites with mass spectrometry, and examined the functional interplay between these cytokinesis regulators and the two protein kinases by genetic and cell biological approaches. We also determined the structural domains of CIF1 required for the interaction with TbPLK and TbAUK1 and the structural domains of TbPLK required for the interaction with CIF1, CIF2, CIF4 and FPRC. Finally, we delineated the order of actions among CIF4, FPRC, TbPLK and other cytokinesis regulators. These results identified CIF1 and CIF2 as substrates of both TbPLK and TbAUK1, and placed CIF4 and FPRC upstream of TbPLK and other cytokinesis regulators in the cytokinesis signalling pathway.

## 2. Results

### 2.1. CIF1 is phosphorylated by TbPLK and TbAUK1 at multiple sites within the intrinsically disordered regions

**Figure 1.**
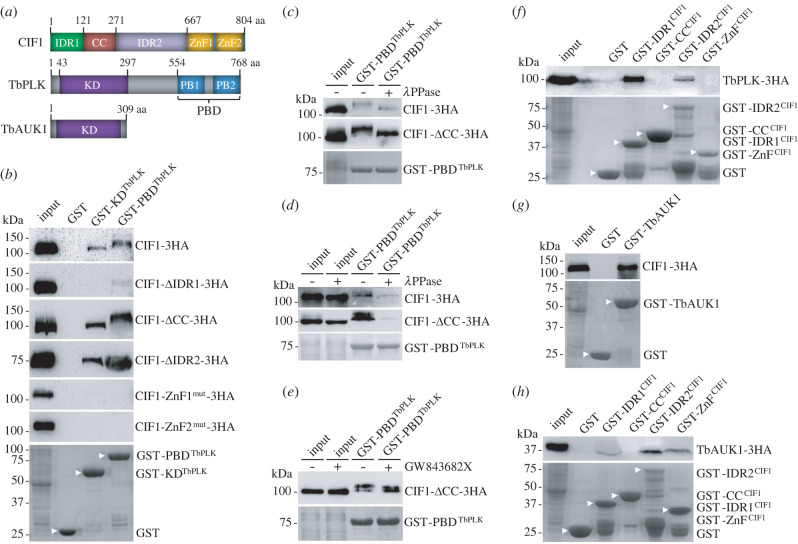
Determination of the structural domains involved in the interaction between CIF1 and TbPLK and between CIF1 and TbAUK1. (*a*) Schematic drawing of the structural domains in CIF1, TbPLK and TbAUK1. IDR, intrinsically disordered region; CC, coiled coil; ZnF, zinc finger; KD, kinase domain; PBD, polo-box domain; PB, polo box. (*b*) *In vitro* pull-down of CIF1 and CIF1 mutants by GST-fused TbPLK structural domains. Triple HA-tagged CIF1 and its mutants were detected by anti-HA antibody. Recombinant GST and GST-fused TbPLK domains (arrowhead) were stained with Coomassie blue. (*c*) Verification of PBD^TbPLK^-precipitated CIF1 and CIF1-ΔCC as phosphorylated forms. Proteins pulled down by GST-PBD^TbPLK^ were treated with Lambda protein phosphatase (*λ*PPase) and then analysed by western blotting with anti-HA antibody. (*d*) Determination of the requirement of CIF1 phosphorylation for binding to GST-PBD^TbPLK^. Cell lysate was treated with *λ*PPase before GST pull-down. (*e*) Determination of the requirement of TbPLK-mediated CIF1 phosphorylation for binding to GST-PBD^TbPLK^. Cells were treated with GW843682X for 16 h and then lysed for GST pull-down. (*f*) *In vitro* pull-down of TbPLK by GST-fused CIF1 domains. TbPLK-3HA was detected by anti-HA antibody. Recombinant GST and GST-fused CIF1 domains (arrowhead) were stained with Coomassie blue. (*g*) *In vitro* pull-down of CIF1 by GST-fused TbAUK1. CIF1-3HA was detected by anti-HA antibody. GST and GST-fused TbAUK1 (arrowhead) were stained with Coomassie blue. (*h*) *In vitro* pull-down of TbAUK1 by GST-fused CIF1 domains. TbAUK1-3HA was detected by anti-HA antibody. Recombinant GST and GST-fused CIF1 domains (arrowhead) were stained with Coomassie blue.

Previous work demonstrated the interaction between CIF1 and the two protein kinases TbPLK and TbAUK1 [16–18,27], but the structural domains involved in their interaction were not determined. CIF1 contains an N-terminal coiled-coil (CC) motif, two C-terminal zinc-finger (ZnF) motifs and two intrinsically disordered regions (IDR) located at the N-terminus and between the CC and the ZnF motifs (figure 1*a*). TbPLK contains an N-terminal kinase domain (KD) and a C-terminal polo-box domain (PBD) composed of two polo-boxes (PB1 and PB2), whereas TbAUK1 contains mostly the KD, with short (approx. 20 residues) unstructured regions at the N- and C-termini (figure 1*a*). We first tested the TbPLK structural domains required for interaction with CIF1 by GST pull-down assay using trypanosome cells expressing triple HA-tagged full-length and structural domain-deleted mutants of CIF1 and purified recombinant GST-fused KD and PBD domains of TbPLK. We found that both the KD and the PBD of TbPLK were able to pull down CIF1, with the PBD pulling down a slower migrating CIF1 protein band on SDS-PAGE (figure 1*b*), suggestive of a potential phosphorylated form of CIF1. Deletion of the IDR1 abolished the pull-down of CIF1 by the KD of TbPLK and severely reduced the amount of CIF1 protein pulled down by the PDB of TbPLK (figure 1*b*), and mutation of either ZnF motifs abolished the pull-down of CIF1 by both the KD and the PBD of TbPLK (figure 1*b*), suggesting that the IDR1 and the ZnF motifs are required for CIF1 interaction with TbPLK. However, deletion of the CC or the IDR2 did not affect the pull-down of CIF1 by both the KD and the PBD of TbPLK (figure 1*b*), although the PBD of TbPLK appeared to pull down a slower migrating CIF1-ΔCC protein band, but not a slower migrating CIF1-ΔIDR2 protein band (figure 1*b*), suggestive of the pull-down of a potential phosphorylated form of CIF1-ΔCC protein. Treatment of the precipitated proteins with Lambda protein phosphatase (*λ*PPase) confirmed that the slower migrating bands of CIF1 and CIF1-ΔCC were both phosphorylated forms (figure 1*c*). To test whether phosphorylation of CIF1 and CIF1-ΔCC is required for binding to the PBD of TbPLK, we treated the trypanosome cell lysate with *λ*PPase and then performed GST pull-down assay. The results showed that upon *λ*PPase treatment, the amount of CIF1 and CIF1-ΔCC proteins precipitated by the PBD of TbPLK was reduced (figure 1*d*), suggesting that the PBD of TbPLK binds to phosphorylated CIF1.

We also tested whether inhibition of TbPLK activity, which was known to cause dephosphorylation of CIF1 in trypanosome cells [16], affected the binding of CIF1 to the PBD of TbPLK. To this end, we treated the cells expressing CIF1-ΔCC with the human Plk1 inhibitor GW843682X [28], which also inhibits TbPLK activity *in vitro* and *in vivo* [15], and then performed GST pull-down assay. The results showed that upon GW843682X treatment, CIF1-ΔCC pulled down by the PBD of TbPLK was partially dephosphorylated, but its amount was unchanged (figure 1*e*), suggesting that TbPLK-mediated CIF1 phosphorylation is not required for the association of CIF1 with the PBD of TbPLK. We next performed reciprocal GST pull-down assay, and we found that both IDR1 and IDR2, but not the CC and the ZnF, were able to pull down TbPLK (figure 1*f*). The inability of pulling down TbPLK by the ZnF motifs of CIF1 (figure 1*f*) and the mutation of the ZnF motifs disrupting the pull-down of CIF1 by TbPLK (figure 1*b*) suggest that the ZnF motifs do not bind to TbPLK and that the mutation of ZnF motifs probably affects the overall CIF1 structure, which impairs the interaction between CIF1 and TbPLK. We next tested the interaction between TbAUK1 and CIF1 by GST pull-down, and we found that TbAUK1 was able to pull down CIF1 (figure 1*g*) and that the two IDRs and the ZnF motifs, but not the CC motif, were able to pull down TbAUK1 (figure 1*h*), demonstrating that TbAUK1 and CIF1 interact *in vitro* and that multiple domains of CIF1 probably make contact with TbAUK1.

**Figure 2.**
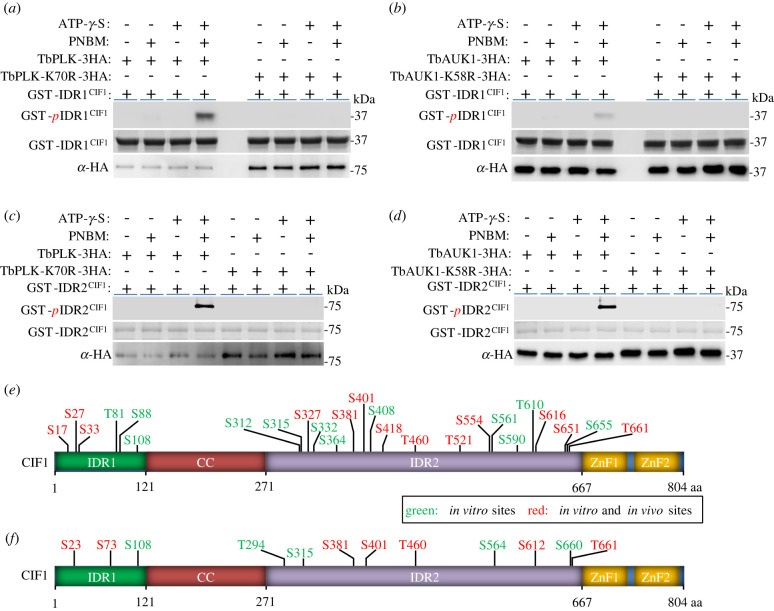
CIF1 is phosphorylated by TbPLK and TbAUK1. (*a*) *In vitro* kinase assay with TbPLK and GST-IDR1^CIF1^ using the thiophosphorylation method. Thiophosphorylated GST-IDR1^CIF1^ was detected by anti-ThioP antibody, and TbPLK-3HA and TbPLK-K70R-3HA were detected by anti-HA antibody. GST- IDR1^CIF1^ was stained with Coomassie blue. PNBM, *p*-nitrobenzylmesylate. (*b*) *In vitro* kinase assay with TbAUK1 and GST-IDR1^CIF1^ using the thiophosphorylation method. Thiophosphorylated GST-IDR1^CIF1^ was detected by anti-ThioP antibody, and TbAUK1-3HA and TbAUK1-K58R-3HA were detected by anti-HA antibody. (*c*) *In vitro* kinase assay with TbPLK and GST-IDR2^CIF1^ using the thiophosphorylation method as described in *a*. (*d*) *In vitro* kinase assay with TbAUK1 and GST-IDR2^CIF1^ using the thiophosphorylation method as described in *b*. (*e*) *In vitro* TbPLK phosphosites on CIF1. The sites highlighted in red were previously reported as *in vivo* phosphosites. (*f*) *In vitro* TbAUK1 phosphosites on CIF1. The sites highlighted in red were previously reported as *in vivo* phosphosites.

Recent phosphoproteomics studies identified 34 *in vivo* phosphosites in CIF1 [29–31], of which 10 phosphosites locate in the IDR1 and 24 phosphosites locate in the IDR2 (electronic supplementary material, figure S1). Because CIF1 interacts with TbPLK and TbAUK1 (figure 1) and inhibition of TbPLK and TbAUK1 kinase activity abolishes CIF1 phosphorylation [16], we attempted to test whether CIF1 is a substrate of TbPLK and TbAUK1. Using purified recombinant GST-IDR1^CIF1^ and GST-IDR2^CIF1^ as substrates, we carried out *in vitro* kinase assay with TbPLK-3HA and TbAUK1-3HA immunoprecipitated from trypanosome cell lysate using the thiophosphorylation method [32]. To rule out the non-specific phosphorylation by any potential co-immunoprecipitated protein kinase(s), we immunoprecipitated the kinase-dead mutants of TbPLK and TbAUK1, TbPLK-K70R-3HA and TbAUK1-K58R-3HA, for *in vitro* kinase assay. The results showed that both TbPLK and TbAUK1, but not TbPLK-K70R and TbAUK1-K58R, were able to thiophosphorylate GST-fused IDR1 and IDR2 of CIF1 (figure 2*a–d*), demonstrating that CIF1 is an *in vitro* substrate of both TbPLK and TbAUK1.

We next performed regular *in vitro* kinase assay followed by mass spectrometry (LC-MS/MS), and we identified 25 serine and threonine residues as TbPLK phosphosites, of which 6 sites located in the IDR1 and 19 sites located in the IDR2 (figure 2*e*, green and red; electronic supplementary material, figure S2). Among the 25 TbPLK phosphosites, 13 sites were previously reported as *in vivo* phosphosites, of which three sites located in the IDR1 and 10 sites located in the IDR2 (figure 2*e*, red). Among the TbPLK phosphosites, three sites, Ser-33, Ser-327 and Ser-651, were predicted to be consensus PLK phosphorylation sites using the group-based phosphorylation scoring (GPS) algorithm [33]. We also identified 12 serine and threonine residues as TbAUK1 phosphosites, of which three sites located in the IDR1 and nine sites located in the IDR2 (figure 2*f*, green and red; electronic supplementary material, figure S3). Seven out of the 12 TbAUK1 phosphosites were previous reported as *in vivo* phosphosites, of which two sites located in the IDR1 and five sites located in the IDR2 (figure 2*f*, red). Among the TbAUK1 phosphosites, six sites, Ser-23, Ser-73, Thr-294, Ser-315, Ser-381 and Ser-660, were predicted to be consensus Aurora B kinase phosphorylation sites by the GPS algorithm. These results confirmed CIF1 as a substrate of TbPLK and TbAUK1, and identified multiple TbPLK- and TbAUK1-phosphorylated sites within the two IDR sequences of CIF1, some of which are potential *in vivo* phosphosites of TbPLK and TbAUK1. Notable, four *in vivo* phosphosites, Ser-381, Ser-401, Thr-460 and Thr-661, and two *in vitro* phosphosites, Ser-108 and Ser-315, were found to be phosphorylated by both TbPLK and TbAUK1 *in vitro* (figure 2*e,f*), suggesting that these sites might be phosphorylated by TbPLK and TbAUK1 during early and late cell cycle stages, respectively.

### 2.2. The intrinsically disordered regions of CIF1 are required for CIF1 function in cytokinesis

**Figure 3.**
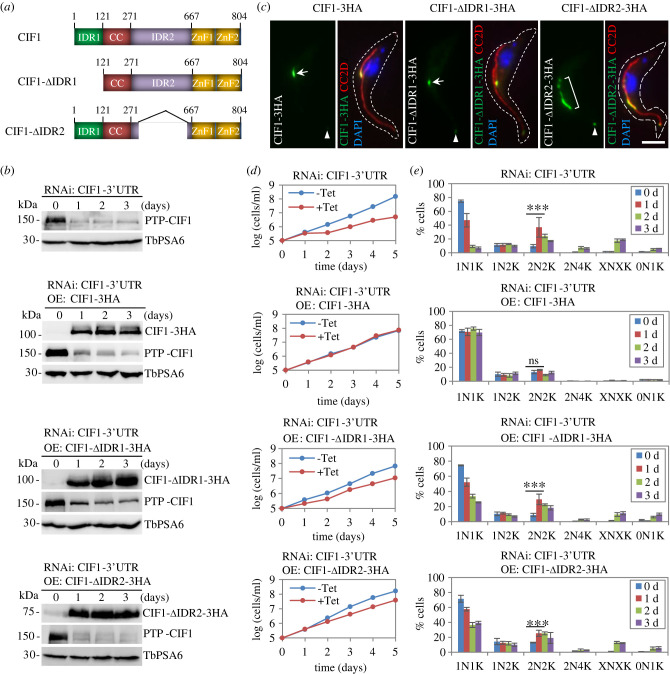
The two intrinsically disordered regions of CIF1 are required for CIF1 function. (*a*) Schematic illustration of the full-length and the IDR-deletion mutants of CIF1 used for genetic complementation. (*b*) Western blotting to monitor CIF1 knockdown by CIF1-3′UTR RNAi and ectopic expression of triple HA-tagged wild-type and IDR-deletion mutants of CIF1. Endogenous PTP-tagged CIF1 was detected by anti-Protein A antibody, and ectopic triple HA-tagged CIF1 and its mutants were detected by anti-HA antibody. TbPSA6 served as a loading control. (*c*) Subcellular localization of ectopically expressed triple HA-tagged CIF1 and IDR-deletion mutants. The triple HA-tagged CIF1 and IDR-deletion mutants were co-immunostained with FITC conjugated anti-HA antibody (green) and anti-CC2D antibody (red) and counterstained with DAPI for DNA (blue). Arrow indicates CIF1 and CIF1-ΔIDR1 signal at the new FAZ tip, bracket outlines the CIF1-ΔIDR2 signal at the anterior one-third part of the new FAZ, and arrowhead indicates the fluorescence signal of wild-type and IDR-deletion mutants of CIF1 at the old FAZ tip. Scale bar: 5 µm. (*d*) Growth curves of CIF1-3′UTR RNAi cell line and CIF1-3′UTR RNAi cell lines expressing wild-type and IDR-deletion mutants of CIF1. (*e*) Effect of IDR-deletion on cell cycle progression. Shown are the counting of cells with different numbers of nuclei (N) and kinetoplasts (K) before and after RNAi induction and ectopic overexpression of wild-type and IDR-deletion mutants of CIF1. Two hundred cells for each time point were counted, and error bars indicate s.d. (*n* = 3). ***, *p* < 0.001; ns, no statistical significance.

**Figure 4.**
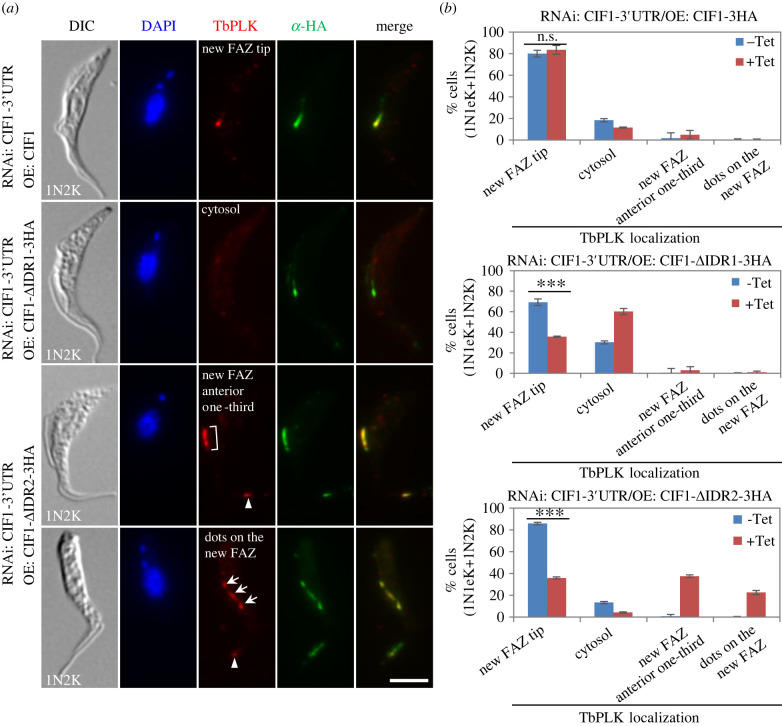
Interaction with the IDR1 of CIF1 is required for TbPLK localization to the new FAZ tip. (*a*) Localization of TbPLK in CIF1-3′UTR RNAi cell lines expressing wild-type and IDR-deletion mutants of CIF1. TbPLK was detected by anti-TbPLK antibody, and triple HA-tagged wild-type and mutants of CIF1 were detected by FITC-conjugated anti-HA antibody. Bracket outlines TbPLK signal at the anterior one-third part of the new FAZ, arrow indicates dotted TbPLK signal at the new FAZ, and arrowhead indicates TbPLK signal at the old FAZ tip. Scale bar: 5 µm. (*b*) Quantification of TbPLK localization in CIF1-3′UTR RNAi cell line expressing wild-type and IDR-deletion mutants of CIF1. Two hundred cells for the S-phase (1N1eK, eK: elongated kinetoplast) and the G2 and early mitotic phases (1N2K) were counted for each treatment (−Tet and +Tet), and the results were presented as mean percentage ± s.d. (*n* = 3). ***, *p* < 0.001, ns, no statistical significance.

The involvement of the two IDRs of CIF1 in the interaction with TbPLK and TbAUK1 and the extensive TbPLK- and TbAUK1-mediated phosphorylation within the two IDRs of CIF1 (figure 2) prompted us to investigate the requirement of the two IDRs for CIF1 function by genetic complementation. To this end, we deleted the entire IDR1 or the phosphosite-containing region of the IDR2 to make an IDR1-deletion mutant or an IDR2-deletion mutant of CIF1, respectively (figure 3*a*), and then expressed the triple HA-tagged CIF1-ΔIDR1 and CIF1-ΔIDR2 in the CIF1-3′UTR RNAi cell line. Ectopic expression of CIF1-ΔIDR1 and CIF1-ΔIDR2 and knockdown of the endogenous PTP-CIF1 were confirmed by western blotting (figure 3*b*). Ectopic expression of 3HA-tagged CIF1 in CIF1-3′UTR RNAi cell line was reported previously [34], and this RNAi complementation cell line was used in the current work (figure 3*b*). Immunofluorescence microscopy showed that CIF1-ΔIDR1 was localized to the new FAZ tip (figure 3*c*, arrow), similar to the wild-type CIF1 (figure 3*c*, arrow), whereas CIF1-ΔIDR2 appeared to be spread onto one-third length of the new FAZ in the anterior region (hereafter referred to as new FAZ anterior one-third), either as a continuous line (figure 3*c*, bracket) or as multiple dots (figure 4*a*). Moreover, at the old FAZ tip, the fluorescence signal of CIF1-ΔIDR2 appeared to be stronger than that of the wild-type CIF1 and CIF1-ΔIDR1 (figure 3*c*, arrowhead). These results suggest that deletion of IDR2 somewhat disrupted the localization of CIF1. Further, while expression of wild-type CIF1 fully complemented the growth defects of the CIF1-3′UTR RNAi cells, as reported previously [34], expression of either of the two IDR-deletion mutants of CIF1 was unable to rescue the growth defects caused by CIF1-3′UTR RNAi (figure 3*d*). Analysis of the effect of IDR-deletion on cell cycle progression by counting the cells with different numbers of nuclei (N) and kinetoplasts (K) showed an initial increase of bi-nucleated (2N2K) cells after 24 h and subsequent accumulation of multi-nucleated (XNXK, X > 2) cells after 48 h, whereas expression of wild-type CIF1 restored cell cycle progression (figure 3*e*). These results demonstrated that both IDRs are required for CIF1 function in cytokinesis.

### 2.3. Interaction with the IDR1 of CIF1 is required for TbPLK localization to the new FAZ tip

We previously reported that depletion of TbPLK and inhibition of TbPLK activity disrupted CIF1 localization at the new FAZ tip, and that depletion of CIF1 impaired TbPLK localization at the new FAZ tip [16,20]. However, it remains unclear whether the interaction with CIF1 is required for TbPLK localization to the new FAZ tip. To test this possibility, we examined the localization of TbPLK in CIF1-3′UTR RNAi cell lines expressing wild-type or each of the two IDR-deletion mutants of CIF1. In the CIF1-3′UTR RNAi cell line expressing CIF1, TbPLK was found to localize to the new FAZ tip, similar to that in the non-induced control cells (figure 4*a*,*b*). In the CIF1-3′UTR RNAi cell line expressing CIF1-ΔIDR1, however, those cells with TbPLK localized at the new FAZ tip decreased from approximately 70% to approximately 35% after tetracycline induction, although CIF1-ΔIDR1 remained to localize to the new FAZ tip (figure 4*a*,*b*). Given that deletion of IDR1 severely impaired CIF1 interaction with TbPLK (figure 1*b*), it suggests that interaction with the IDR1 of CIF1 is required for localizing TbPLK to the new FAZ tip. In the CIF1-3′UTR RNAi cell line expressing CIF1-ΔIDR2, after tetracycline induction the cells with TbPLK localized at the new FAZ tip decreased from approximately 86% to approximately 36%, whereas the cells with TbPLK localized at the new FAZ anterior one-third (figure 4*a*, bracket) or with TbPLK detected as multiple dots on the new FAZ (figure 4*a*, arrow) increased to approximately 38% and approximately 23%, respectively (figure 4*a*,*b*). It was also noted that TbPLK was additionally detectable at the old FAZ tip in the cells expressing CIF1-ΔIDR2 (figure 4*a*, arrowhead). This pattern of TbPLK localization is similar to that of CIF1-ΔIDR2 (figure 4*a*), probably because the two proteins are still capable of forming a complex (figure 1*b*) and, hence, are co-localized together. These results suggest that TbPLK localization to the new FAZ tip depends on the interaction with CIF1, which is in agreement with the finding made in other eukaryotes that PLK substrates mediate PLK localization to specific subcellular structures [35].

### 2.4. TbAUK1 activity is required for maintaining CIF1 at the new FAZ tip from late mitosis

**Figure 5.**
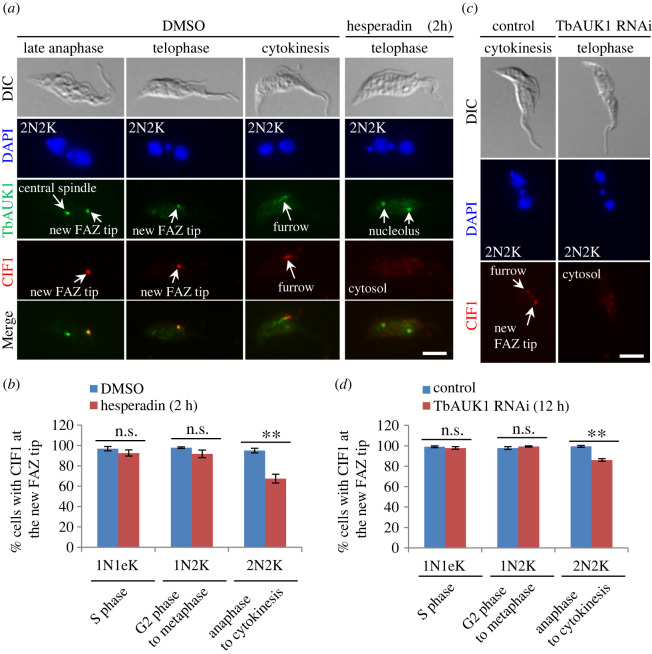
TbAUK1 is required for maintaining CIF1 at the new FAZ tip from late mitosis. (*a*) Effect of TbAUK1 inhibition on CIF1 localization. Cells expressing endogenously triple HA-tagged TbAUK1 were treated with Hesperadin and co-immunostained with anti-CIF1 antibody and FITC-conjugated anti-HA antibody. Scale bar: 5 µm. (*b*) Quantification of cells with CIF1 localized at the new FAZ tip in DMSO-treated and Hesperadin-treated cells. Two hundred cells were counted for each cell type and each treatment, and the results were presented as mean percentage ± s.d. (*n* = 3). **, *p* < 0.01; ns*,* no statistical significance. (*c*) Effect of TbAUK1 depletion on CIF1 localization. TbAUK1 RNAi was inducted for 12 h, and cells were immunostained with anti-CIF1 antibody. Scale bar: 5 µm. (*d*) Quantification of cells with CIF1 localized at the new FAZ tip in non-induced control and TbAUK1 RNAi-induced cells. Two hundred cells were counted for each cell type and each cell line, and the results were presented as mean percentage ± s.d. (*n* = 3). **, *p* < 0.01; ns*,* no statistical significance.

We previously reported that inhibition of TbAUK1 activity with the Aurora B kinase inhibitor Hesperadin [36,37] and knockdown of TbAUK1 by RNAi had no effect on CIF1 localization in cells that had been arrested before mitotic onset [16]. Given that CIF1 and TbAUK1 only co-localize to the new FAZ tip after late mitosis [16], it suggests that TbAUK1 may phosphorylate CIF1 at the new FAZ tip from late mitosis and onward, but whether TbAUK1 activity is required for CIF1 localization from late mitosis remains unclear. Given that prolonged inhibition of TbAUK1 by Hesperadin and depletion of TbAUK1 by RNAi arrested cells at the G2/M transition, producing mostly 1N2K cells [13,14], we thus tested the effect of TbAUK1 inhibition and knockdown for a shorter time on the localization of CIF1 during late mitosis in 2N2K cells. We treated cells with Hesperadin for 2 h, and then examined the localization of CIF1 and endogenous 3HA-tagged TbAUK1 by co-immunofluorescence microscopy. In the cells treated with DMSO, TbAUK1 was detected at the central spindle and the new FAZ tip in anaphase cells, at the new FAZ tip in telophase cells, and at the cleavage furrow in cells undergoing cytokinesis (figure 5*a*), as reported previously [38]. CIF1 co-localized with TbAUK1 at the new FAZ tip during late anaphase and telophase and at the cleavage furrow during cytokinesis (figure 5*a*). However, after Hesperadin treatment for 2 h, the 2N2K cells with CIF1 localized at the new FAZ tip decreased from approximately 95% to approximately 67% (figure 5*a*,*b*). In those approximately 28% 2N2K cells that had lost CIF1 signal at the new FAZ tip, TbAUK1 was mis-localized to the nucleolus (figure 5*a*), consistent with our previous report that TbAUK1 activity is required for TbAUK1 localization to the new FAZ tip during mitosis [10]. Localization of CIF1 to the new FAZ tip from S phase until metaphase was, however, not affected by Hesperadin treatment (figure 5*b*), which is in agreement with our previous results that TbAUK1 activity is not required for CIF1 localization before metaphase [16]. Thus, TbAUK1 activity is not required for targeting CIF1 to the new FAZ tip during S phase, but is necessary for maintaining CIF1 at the new FAZ tip from late anaphase to cytokinesis.

To corroborate the results obtained from the TbAUK1 inhibition experiments, we examined the localization of CIF1 in TbAUK1 RNAi cells. To avoid rapid arrest of cells at the G2/M-phase junction by TbAUK1 RNAi, we induced RNAi for only 12 h, and then immunostained the cells with the anti-CIF1 antibody to examine CIF1 localization. The results showed that knockdown of TbAUK1 did not affect CIF1 localization in the cells at cell cycle stages from S phase to metaphase, but the anaphase and telophase (2N2K) cells with CIF1 at the new FAZ tip decreased from approximately 99% to approximately 86% (figure 5*c*,*d*). This result recapitulated the Hesperadin-treatment result and demonstrated that TbAUK1 is required for maintaining CIF1 at the new FAZ tip after late mitosis.

### 2.5. CIF2 is phosphorylated by TbPLK and TbAUK1 in the intrinsically disordered region

Using the recently generated anti-CIF2 polyclonal antibody [39], we determined CIF2 localization in the procyclic form of *T. brucei* by immunofluorescence microscopy. The results showed that CIF2 localized to the new FAZ tip from S phase until cytokinesis and additionally localized to the cleavage furrow during cytokinesis (electronic supplementary material, figure S4*a*). Previously, we showed that the C-terminally triple HA-tagged CIF2 was localized to the new FAZ during S phase only [19]. The discrepancy suggests that epitope tagging of CIF2 at the C-terminus restricted its localization to the new FAZ tip after S phase. However, the N-terminally Myc-tagged CIF2 was localized normally from S phase to cytokinesis (electronic supplementary material, figure S4*b*), and knockdown of CIF2 by RNAi depleted the CIF2 signal detected by anti-CIF2 antibody and anti-Myc antibody (electronic supplementary material, figure S4*c*), which validated the anti-CIF2 antibody for detecting native CIF2 protein in trypanosome cells. Previously we showed that CIF1 and CIF2 form a complex *in vivo* in trypanosomes [19], and co-immunofluorescence microscopy showed that CIF2 and CIF1-3HA co-localize to the new FAZ tip from S phase to cytokinesis and, additionally, to the cleavage furrow during cytokinesis (electronic supplementary material, figure S4*d*).

**Figure 6.**
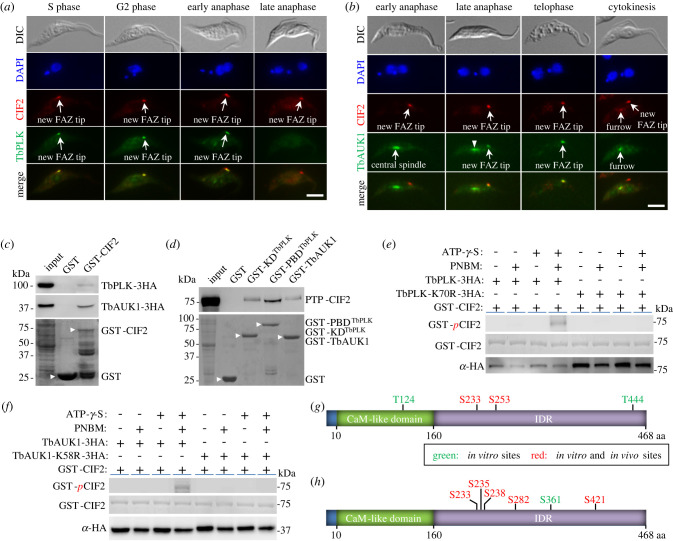
CIF2 is phosphorylated by TbPLK and TbAUK1. (*a*) Co-localization of CIF2 and TbPLK during early cell cycle stages. Endogenously triple HA-tagged TbPLK was detected by FITC-conjugated anti-HA antibody, and CIF2 was detected by anti-CIF2 antibody. Scale bar: 5 µm. (*b*) Co-localization of CIF2 and TbAUK1 during late cell cycle stages. Endogenously triple HA-tagged TbAUK1 was detected by FITC-conjugated anti-HA antibody, and CIF2 was detected by anti-CIF2 antibody. Arrowhead indicates TbAUK1 signal on the central spindle during late anaphase. Scale bar: 5 µm. (*c*) *In vitro* pull-down of TbPLK and TbAUK1 by GST-fused CIF2. TbPLK-3HA and TbAUK1-3HA were detected by anti-HA antibody, and recombinant GST and GST-CIF2 (arrowhead) were stained with Coomassie blue. (*d*) *In vitro* pull-down of CIF2 by GST-fused TbPLK structural domains and GST-TbAUK1. PTP-CIF2 was detected by anti-Protein A antibody, and recombinant GST and GST-fusion proteins (arrowhead) were stained with Coomassie blue. (*e*) *In vitro* kinase assay with TbPLK and GST-CIF2 using the thiophosphorylation method. Thiophosphorylated GST-CIF2 was detected by anti-ThioP antibody, and TbPLK-3HA and TbPLK-K70R-3HA were detected by anti-HA antibody. GST-CIF2 was stained with Coomassie blue. PNBM, *p*-nitrobenzylmesylate. (*f*) *In vitro* kinase assay with TbAUK1 and GST-CIF2 using the thiophosphorylation method. Thiophosphorylated GST-CIF2 was detected by anti-ThioP antibody, and TbAUK1-3HA and TbAUK1-K58R-3HA were detected by anti-HA antibody. GST-CIF2 was stained with Coomassie blue. (*g*) *In vitro* TbPLK phosphosites on CIF2. The sites highlighted in red were previously reported as *in vivo* phosphosites. (*h*) *In vitro* TbAUK1 phosphosites on CIF2. The sites highlighted in red were previously reported as *in vivo* phosphosites.

We investigated the potential co-localization of CIF2 with TbPLK and TbAUK1, which were endogenously tagged with a triple HA epitope, by immunofluorescence microscopy using anti-CIF2 antibody and anti-HA antibody. We found that CIF2 and TbPLK co-localized at the new FAZ tip from S phase to early anaphase (figure 6*a*), and that CIF2 and TbAUK1 co-localized at the new FAZ tip from late anaphase to telophase and at the cleavage furrow during cytokinesis (figure 6*b*). These results suggest that TbPLK and TbAUK1 might regulate CIF2 during early and late cell cycle stages, respectively. To test whether CIF2 interacts with the two protein kinases, we performed *in vitro* GST pull-down assay, and the results showed that recombinant GST-fused CIF2 was able to pull down both TbPLK and TbAUK1 from cell lysate (figure 6*c*). Reciprocal pull-down assay using recombinant GST-fused KD and PBD of TbPLK and GST-fused TbAUK1 showed that the KD and the PBD of TbPLK and TbAUK1 were all able to pull down CIF2 from cell lysate (figure 6*d*).

Previous phosphoproteomics studies identified 20 *in vivo* phosphosites on CIF2 [29–31], 18 of which are located within the C-terminal IDR (electronic supplementary material, figure S5). The pull-down of CIF2 by TbPLK and TbAUK1 (figure 6*c*,*d*) suggests that CIF2 might be phosphorylated by the two protein kinases. To test this possibility, we carried out *in vitro* kinase assay using recombinant GST-CIF2 purified from bacteria and triple HA-tagged wild-type and kinase-dead mutants of TbPLK and TbAUK1 immunoprecipitated from *T. brucei* by the thiophosphorylation method. The results showed that both TbPLK and TbAUK1, but not the kinase-dead mutants TbPLK-K70R and TbAUK1-K58R, were able to thiophosphorylate GST-fused CIF2 (figure 6*e*,*f*). We next performed regular *in vitro* kinase assays and analysed the phosphorylated CIF2 by mass spectrometry. These assays identified two threonine residues, Thr-124 and Thr-444, and two serine residues, Ser-233 and Ser-253, on CIF2 as TbPLK phosphosites (figure 6*g*, green and red; electronic supplementary material, figure S6). Ser-233 and Ser-253 are among those *in vivo* phosphosites identified previously (figure 6*g*, red), and Ser-253 was predicted to be a consensus PLK phosphosite by the GPS algorithm, suggesting that CIF2 is a potential *in vivo* substrate of TbPLK. We also identified six *in vitro* TbAUK1 phosphosites on CIF2 (figure 6*h*, green and red; electronic supplementary material, figure S7), of which five sites are among the previously identified *in vivo* phosphosites (figure 6*h*, red), and Ser-421 was predicted to be a consensus Aurora B kinase phosphosite by the GPS algorithm, suggesting that CIF2 is also a potential *in vivo* substrate of TbAUK1. All six TbAUK1 phosphosites were located within the C-terminal IDR of CIF2 (figure 6*h*). Notably, Ser-233 was phosphorylated by both TbPLK and TbAUK1 *in vitro* (figure 6*g*,*h*), suggesting that CIF2 may be phosphorylated on this site by TbPLK during early cell cycle stages and by TbAUK1 during late cell cycle stages.

### 2.6. TbPLK activity is required for maintaining CIF2 at the new FAZ tip from G2 phase

**Figure 7.**
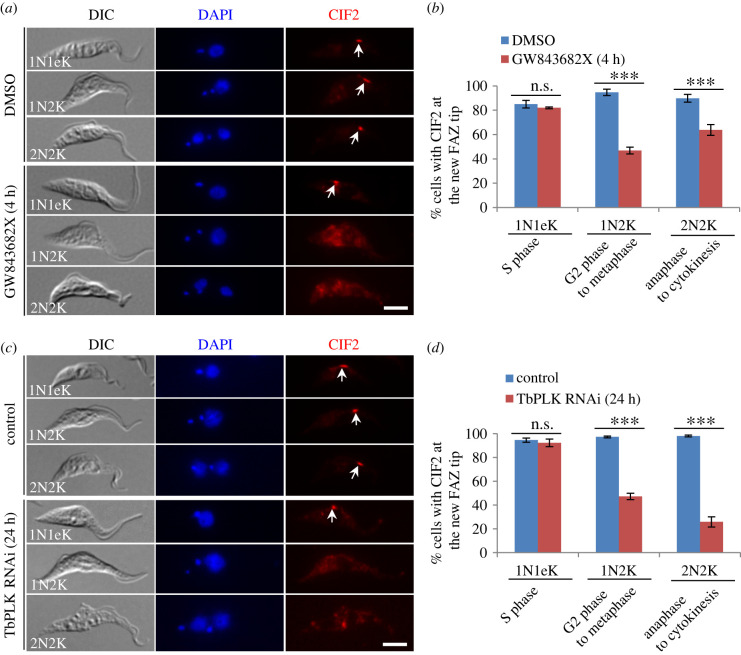
TbPLK is required for maintaining CIF2 at the new FAZ tip from G2 phase and onward. (*a*) Effect of TbPLK inhibition on CIF2 localization. Cells were treated with GW843682X, and immunostained with anti-CIF2 antibody to detect CIF2 (arrows). Scale bar: 5 µm. (*b*) Quantification of cells with CIF2 localized at the new FAZ tip (arrows) in DMSO-treated and GW843682X-treated cells at different cell cycle stages. Two hundred cells were counted for each cell type and each treatment, and the results were presented as mean percentage ± s.d. (*n* = 3). ***, *p* < 0.001; ns*,* no statistical significance. (*c*) Effect of TbPLK depletion on CIF2 localization. CIF2 was detected by anti-CIF2 antibody. Arrow indicates CIF2 signal. Scale bar: 5 µm. (*d*) Quantification of cells with CIF2 localized at the new FAZ tip in non-induced control and TbPLK RNAi-induced cells. Two hundred cells were counted for each cell type and each cell line, and the results were presented as mean percentage ± s.d. (*n* = 3). ***, *p* < 0.001; ns*,* no statistical significance.

The identification of CIF2 as an *in vitro* substrate of TbPLK prompted us to investigate the potential effects of TbPLK inhibition and depletion on the localization of CIF2. To this end, we first tested the inhibition of TbPLK on CIF2 localization by immunofluorescence microscopy. To minimize the inhibitory effects of TbPLK deficiency on the formation of the new FAZ as well as any potential secondary effects, *T. brucei* cells were treated with GW843682X for 4 h, and then immunostained with anti-CIF2 antibody. Cells at different cell cycle stages were counted for the presence of CIF2 fluorescence signal at the new FAZ tip. The results showed that inhibition of TbPLK activity by GW843682X did not significantly affect CIF2 localization in 1N1eK cells, but reduced the percentage of CIF2-positive 1N2K cells and 2N2K cells by approximately 48% and approximately 26%, respectively (figure 7*a*,*b*). These results suggest that TbPLK activity is required for CIF2 localization in 1N2K and 2N2K cells. To corroborate the results obtained from the GW843682X-mediated TbPLK inhibition experiments, we investigated the effect of TbPLK knockdown on CIF2 localization during different cell cycle stages. RNAi of TbPLK was induced for 24 h to minimize the effects of TbPLK depletion on FAZ assembly so as to prevent the indirect effects of FAZ assembly defects on CIF2 localization. The results showed that knockdown of TbPLK did not affect the localization of CIF2 in 1N1eK cells, but caused a significant decrease of CIF2-positive 1N2K cells and 2N2K cells by approximately 50% and approximately 72%, respectively (figure 7*c*,*d*), which recapitulated the results obtained from the inhibition of TbPLK activity by GW843682X treatment. However, it was noted that inhibition of TbPLK activity exerted stronger effects on CIF2 localization in 1N2K cells than in 2N2K cells, whereas knockdown of TbPLK exerted stronger effects on CIF2 localization in 2N2K cells than in 1N2K cells (figure 7*b*,*d*). Such different effects might be due to the difference in the durations of TbPLK inhibition (4 h) and TbPLK knockdown (24 h), the latter of which affected cell cycle progression to accumulate 2N2K cells [16]. Another possibility is that TbPLK knockdown disrupted TbAUK1 localization [16], and because TbAUK1 also maintains CIF2 localization in 2N2K cells (see below), the disrupted TbAUK1 localization exerted additional effects on CIF2 localization in the TbPLK-deficient 2N2K cells. Nonetheless, these results demonstrated that TbPLK activity inhibition and TbPLK knockdown both impaired CIF2 localization in 1N2K and 2N2K cells. Finally, since CIF2 started to localize to the new FAZ tip from S phase (electronic supplementary material, figure S4), during which it was not affected by TbPLK depletion and inhibition (figure 7), it suggests that TbPLK is unlikely to be involved in recruiting CIF2 to the new FAZ tip, but rather is required for maintaining CIF2 at the new FAZ tip from G2 phase to cytokinesis.

### 2.7. TbAUK1 activity is required for maintaining CIF2 at the new FAZ tip from late mitosis

**Figure 8.**
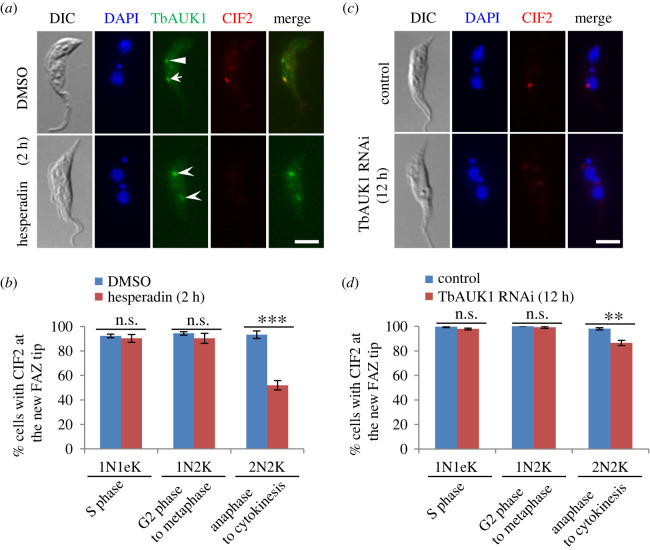
TbAUK1 is required for maintaining CIF2 at the new FAZ tip from late mitosis to cytokinesis. (*a*) Effect of TbAUK1 inhibition on CIF2 localization. Cells expressing endogenously 3HA-tagged TbAUK1 were treated with Hesperadin and then immunostained with FITC-conjugated anti-HA antibody and anti-CIF2 antibody. Arrow indicates TbAUK1 at the new FAZ tip, arrowhead indicates TbAUK1 at the central spindle, and open arrowhead indicates TbAUK1 in the nucleolus. Scale bar: 5 µm. (*b*) Quantification of cells with CIF2 localized at the new FAZ tip in DMSO-treated and Hesperadin-treated cells. Two hundred cells were counted for each cell type and each treatment, and the results were presented as mean percentage ± s.d. (*n* = 3). ***, *p* < 0.001; ns*,* no statistical significance. (*c*) Effect of TbAUK1 depletion on CIF2 localization. CIF2 was detected by anti-CIF2 antibody. Scale bar: 5 µm. (*d*) Quantification of cells with CIF2 localized at the new FAZ tip in non-induced control and TbAUK1 RNAi-induced cells. Two hundred cells were counted for each cell type and each cell line, and the results were presented as mean percentage ± s.d. (*n* = 3). **, *p* < 0.01; ns*,* no statistical significance.

Because CIF2 is a substrate of TbAUK1 and they co-localize to the new FAZ tip from late anaphase to cytokinesis (figure 6), we wondered whether TbAUK1 activity was required for maintaining CIF2 at the new FAZ tip during late mitotic phases. To this end, we inhibited TbAUK1 activity by Hesperadin treatment and depleted TbAUK1 by RNAi, and then examined the effects on CIF2 localization during different cell cycle stages. Cells were treated with Hesperadin for 2 h and TbAUK1 RNAi was induced for 12 h to avoid the arrest of cells at the G2/M boundary of the cell cycle. Immunofluorescence microscopy with anti-CIF2 antibody showed that inhibition of TbAUK1 activity by Hesperadin and knockdown of TbAUK1 by RNAi both caused the decrease of the CIF2-positive 2N2K cells by approximately 41% and approximately 13%, respectively, but did not affect CIF2 localization in 1N1eK and 1N2K cells (figure 8). The effects exerted by TbAUK1 inhibition and depletion on CIF2 localization were similar to that on CIF1 localization (figure 5), likely because CIF1 and CIF2 form a functionally interdependent protein complex [19] and, hence, they are regulated as a single unit by TbAUK1. These results suggest that TbAUK1 is required for maintaining CIF2 at the new FAZ tip from late mitosis to cytokinesis.

### 2.8. CIF4 and FPRC function independently and upstream of TbPLK in the cytokinesis pathway

**Figure 9.**
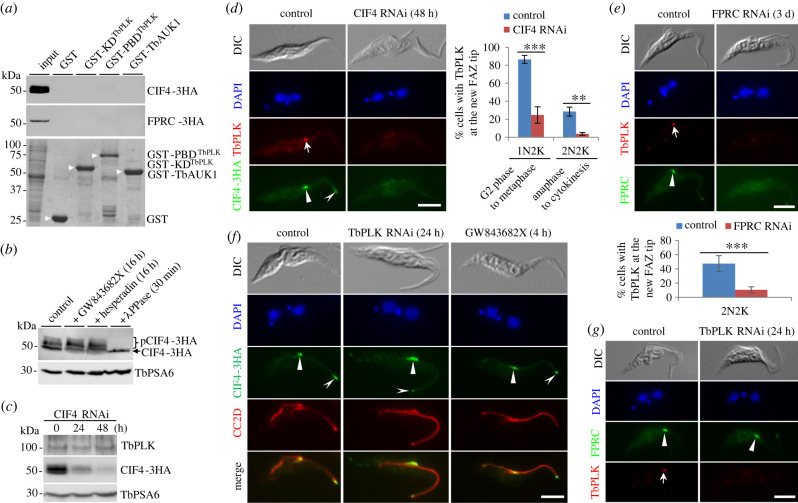
CIF4 and FPRC function independently and upstream of TbPLK in the cytokinesis pathway. (*a*) *In vitro* GST pull-down to test the interaction of CIF4 and FPRC with TbPLK and TbAUK1. CIF4-3HA and FPRC-3HA were detected by anti-HA antibody. Recombinant GST and GST-fusion proteins (arrowhead) were stained with Coomassie blue. (*b*) CIF4 is phosphorylated *in vivo* in *T. brucei* cells independently of TbPLK and TbAUK1 activities. Cells expressing CIF4-3HA were treated with GW843682X or Hesperadin, and cell lysate was treated with Lambda protein phosphatase (*λ*PPase). pCIF4-3HA, phosphorylated CIF4-3HA. TbPSA6 served as a loading control. (*c*) Western blotting to monitor the protein levels of TbPLK and CIF4 before and after CIF4 RNAi induction. TbPLK was detected by anti-TbPLK antibody, and endogenous triple HA-tagged CIF4 was detected by anti-HA antibody. TbPSA6 served as a loading control. (*d*) Effect of CIF4 knockdown on TbPLK localization. TbPLK was detected by anti-TbPLK antibody, and endogenous triple HA-tagged CIF4 was detected by FITC-conjugated anti-HA antibody. Arrow indicates TbPLK signal at the new FAZ tip, whereas solid and open arrowheads indicate CIF4-3HA signal at the new and old FAZ tips, respectively. The graph shows the counting of cells with TbPLK localized at the new FAZ tip in non-induced control and CIF4 RNAi-induced cells, presented as mean percentage ± s.d. (*n* = 3). **, *p* < 0.01; ***, *p* < 0.001. Scale bar: 5 µm. (*e*) Effect of TbPLK knockdown and activity inhibition on CIF4 localization. Shown is the immunofluorescence microscopic analyses of endogenous triple HA-tagged CIF4 in cells depleted of TbPLK by RNAi or treated with GW843682X. Cells were co-immunostained with FITC-conjugated anti-HA antibody to detect CIF4-3HA and anti-CC2D antibody to label the FAZ. Solid and open arrowheads indicate CIF4-3HA signal at the new and old FAZ tips, respectively. Scale bar: 5 µm. (*f*) Effect of FPRC knockdown on TbPLK localization. The graph shows the percentages of 2N2K cells with TbPLK localized at the new FAZ tip in non-induced control and FPRC RNAi-induced cells, presented as mean percentage ± s.d. (*n* = 3). ***, *p* < 0.001. Scale bar: 5 µm. (*g*) Effect of TbPLK knockdown on FPRC localization. In (*f*,*g*), the endogenous triple HA-tagged FPRC was detected by anti-HA antibody and TbPLK was detected by anti-TbPLK antibody. Arrow indicates TbPLK signal at the new FAZ tip, whereas arrowhead indicates FPRC-3HA signal at the new FAZ tip.

Cytokinesis initiation in *T. brucei* also requires the trypanosomatid-specific regulators CIF3 [20], CIF4 [25] and FPRC [22,25], in addition to CIF1 and CIF2. We recently reported that CIF3 is a substrate of TbPLK, but not TbAUK1, and it targets TbPLK to the new FAZ tip during S phase [40], whereas TbPLK maintains CIF3 at the new FAZ tip from G2 phase and promotes the formation of the CIF1-CIF3 complex [20]. To test whether CIF4 and FPRC are potential substrates of TbPLK and TbAUK1, we first carried out GST pull-down assays to test whether they interact *in vitro*. The results showed that neither CIF4 nor FPRC was precipitated by the KD and the PBD of TbPLK or by TbAUK1 (figure 9*a*), suggesting that CIF4 and FPRC are not interacting partners of TbPLK and TbAUK1 and, hence, are unlikely to be substrates of TbPLK and TbAUK1. Previous phosphoproteomics studies identified seven phosphosites on CIF4, but no phosphosite on FPRC [29–31]. Western blotting of the lysate of *T. brucei* cells expressing endogenously triple HA-tagged CIF4 detected multiple slower migrating bands of CIF4 (figure 9*b*), suggesting that they may be phosphorylated forms of CIF4. Indeed, treatment of trypanosome cell lysate with Lambda protein phosphatase eliminated those slower migrating bands of CIF4 (figure 9*b*), confirming them as the phosphorylated forms of CIF4. However, treatment of trypanosome cells with GW843682X or Hesperadin did not eliminate any of those slower migrating bands of CIF4 (figure 9*b*), suggesting that these phosphorylated forms of CIF4 are not derived from TbPLK- and TbAUK1-mediated phosphorylation.

The finding that CIF4 and FPRC are not substrates of TbPLK led us to hypothesize that they function independently of and upstream of TbPLK and other TbPLK-regulated cytokinesis regulators, such as CIF1 and CIF2. We previously reported that CIF4 knockdown disrupted CIF1 localization [25] (electronic supplementary material, figure S8*a*), but not vice versa (electronic supplementary material, figure S8*b*), and FPRC knockdown disrupted CIF1 localization, but not vice versa [25]. Further, CIF4 knockdown disrupted FPRC localization [25] (electronic supplementary material, figure S8*c*), but not vice versa (electronic supplementary material, figure S8*d*). These results support the hypothesis that CIF4 functions upstream of FPRC and FPRC acts upstream of CIF1 in the cytokinesis pathway. To test the hypothesis that CIF4 functions upstream of TbPLK, we examined the localization of TbPLK in CIF4 RNAi cells and the localization of CIF4 in TbPLK RNAi cells or TbPLK activity-inhibited cells. Western blotting confirmed the knockdown of CIF4 by RNAi and also showed that the levels of TbPLK were not affected by CIF4 RNAi (figure 9*c*). Immunofluorescence microscopy showed that RNAi of CIF4 caused the decrease of the 1N2K cells and 2N2K cells with TbPLK localized at the new FAZ tip by approximately 62% (from approx. 87% to approx. 25%) and approximately 25% (from approx. 29% to approx. 4%), respectively (figure 9*d*). Because no 1N1eK cells were detectable after CIF4 RNAi, we were unable to examine the localization of TbPLK in the S-phase cells. Nonetheless, these results suggest that CIF4 is required for TbPLK localization. Conversely, we examined the effect of TbPLK knockdown and inhibition on CIF4 localization. Since TbPLK deficiency disrupts the assembly of the new FAZ [41], and hence will indirectly affect CIF4 localization, we co-immunostained the cells with the CC2D antibody to label the FAZ. We found that knockdown of TbPLK or inhibition of TbPLK activity by GW843682X did not affect CIF4 localization in all the examined cells that had an intact new FAZ (figure 9*e*). These results suggest that CIF4 functions upstream of TbPLK in the cytokinesis pathway. Finally, we tested the order of actions between FPRC and TbPLK by examining the knockdown of FPRC on TbPLK localization and the knockdown of TbPLK on FPRC localization. Due to the low efficiency of FPRC knockdown, which did not completely deplete FPRC protein at the new FAZ tip in 1N1eK and 1N2K cells, we focused on the 2N2K cells in which FPRC was completely depleted (figure 9*f*). In these 2N2K cells, TbPLK localization to the new FAZ tip was impaired, causing the 2N2K cells with a positive TbPLK signal reduced from approximately 47% to approximately 10% (figure 9*f*). Conversely, knockdown of TbPLK did not affect FPRC localization to the new FAZ tip in all the cells examined (figure 9*g*). The results from the reciprocal experiments suggest that FPRC functions upstream of TbPLK. Altogether, the analyses of the functional interplay between CIF4 and TbPLK and between FPRC and TbPLK demonstrated that CIF4 and FPRC are upstream of TbPLK in the cytokinesis pathway.

## 3. Discussion

Cytokinesis initiation in the procyclic form of *T. brucei* requires two evolutionarily conserved protein kinases, TbPLK and TbAUK1 [11–14], and a cohort of trypanosomatid-specific regulators, including CIF1, CIF2, CIF3, CIF4, FPRC and KPP1 [16–23,25]. Although previous studies showed that CIF1 phosphorylation was impaired by inhibition and knockdown of TbPLK and TbAUK1 [16,18], direct evidence to confirm CIF1 as a substrate of both protein kinases was not provided. Through *in vitro* GST pull-down and *in vitro* kinase assays followed by mass spectrometry, we confirmed CIF1 and CIF2 as *in vitro* substrates of TbPLK and TbAUK1 and mapped the TbPLK- and TbAUK1-phosphorylated sites to the IDRs of CIF1 and CIF2 (figures 2 and 6). Some of the *in vitro* phosphosites on CIF1 and CIF2 were previously identified as *in vivo* phosphosites [29–31], suggesting that CIF1 and CIF2 are potential *in vivo* substrates of TbPLK and TbAUK1 in *T. brucei* cells. However, GST pull-down assays showed that CIF4 and FPRC, which form a complex in *T. brucei* [25], do not interact with TbPLK and TbAUK1 *in vitro* (figure 9*a*). Further, inhibition of TbPLK and TbAUK1 activity by small-molecule inhibitors did not affect CIF4 phosphorylation (figure 9*b*). These results ruled out the possibility that CIF4 and FPRC are substrates of TbPLK and TbAUK1. Previously, we reported that CIF3 interacts with and is phosphorylated by TbPLK *in vitro*, but it does not interact with TbAUK1 [40]. We also reported that KPP1 interacts with TbPLK *in vitro* and *in vivo* and dephosphorylates TbPLK on Thr-125 [24]. However, it remains unclear whether TbPLK exerts any feedback regulation on KPP1 by phosphorylating KPP1. Moreover, because KPP1 disappears from the new FAZ tip from early mitosis [21] and TbAUK1 localizes to the new FAZ tip from late mitosis [38], it suggests that KPP1 is unlikely to co-localize with TbAUK1 and to function as a substrate of TbAUK1. Taken together, these studies identified three cytokinesis regulators, CIF1, CIF2 and CIF3, as TbPLK substrates and two cytokinesis regulators, CIF1 and CIF2, as TbAUK1 substrates. Since TbPLK and TbAUK1 localize to the new FAZ tip during early and late cell cycle stages, respectively, we speculate that TbPLK may phosphorylate CIF1, CIF2 and CIF3 from S phase to early anaphase and that TbAUK1 may phosphorylate CIF1 and CIF2 from late anaphase to cytokinesis.

Although both IDRs of CIF1 are involved in mediating the interaction of CIF1 with TbPLK, only the deletion of IDR1 severely impairs the interaction with TbPLK (figure 1*b*,*f*) and disrupts the localization of TbPLK to the new FAZ tip during S phase to G2 phase (figure 4). Previously, we showed that inhibition of TbPLK by GW843682X and knockdown of TbPLK by RNAi disrupted CIF1 localization during G2 to mitotic phases, but not during S phase [16,20]. Therefore, as a substrate of TbPLK, CIF1 functions to target TbPLK to the new FAZ tip during early cell cycle stages, and, in turn, TbPLK maintains CIF1 localization at the new FAZ tip from G2 phase to early anaphase. The role of CIF1 as a substrate of TbPLK in targeting TbPLK to the new FAZ tip is in agreement with the role of PLK substrates in other systems reported previously [35]. The role of TbPLK in maintaining CIF1 at the new FAZ tip remains unclear, but our previous findings that TbPLK promotes CIF1-CIF3 complex formation [20] and this complex formation is required for CIF1 localization to the new FAZ tip [40] suggests that TbPLK may perform its role in maintaining CIF1 localization by promoting CIF1-CIF3 complex formation. Further, CIF1 also interacts with and is phosphorylated by TbAUK1 (figures 1 and 2), and is required for TbAUK1 localization to the new FAZ tip from late anaphase [16]. Because TbAUK1 starts to appear at the new FAZ during late anaphase [38], it suggests that CIF1 likely is involved in recruiting TbAUK1 to the new FAZ tip when the cell cycle progresses into late anaphase. It also suggests that CIF1 likely is phosphorylated by TbAUK1 after they co-localize at the new FAZ tip during late anaphase. Further investigations demonstrated the requirement of TbAUK1 activity for maintaining CIF1 localization at the new FAZ tip from late anaphase to cytokinesis (figure 5). Together, there appears to be the interdependence between CIF1 and TbPLK and between CIF1 and TbAUK1 for their localization to the new FAZ tip during different cell cycle stages, highlighting the complexity of the regulatory mechanisms among these cytokinesis regulators.

CIF2 forms a complex with CIF1 through the ZnF motifs of CIF1 and the Calmodulin-like domain of CIF2, and they are interdependent for maintaining protein stability [19,34]. CIF1 and CIF2 co-localize at the new FAZ tip from S phase to cytokinesis and, additionally, at the cleavage furrow during cytokinesis (electronic supplementary material, figure S4*d*). Thus, we hypothesize that the CIF1-CIF2 complex may function as a single unit to promote cytokinesis initiation. Because the CIF1-CIF2 complex localize to the cleavage furrow (electronic supplementary material, figure S4*d*) and knockdown of CIF1 impairs the localization of KLIF [22], an essential regulator of cleavage furrow ingression and cytokinesis completion [23,26], it suggests that CIF1 and CIF2 may also regulate cleavage furrow ingression, although this function cannot be tested by knockdown of CIF1 or CIF2 due to the arrest of cells prior to cytokinesis initiation. Nonetheless, in agreement with the proposed hypothesis, CIF2 also interacts with and is phosphorylated by both TbPLK and TbAUK1, likely during early and late cell cycle stages when it co-localizes with TbPLK and TbAUK1, respectively (figure 6*a*,*b*). Moreover, the results from previous and current work demonstrated the interdependence between CIF2 and TbPLK and between CIF2 and TbAUK1 for their localization to the new FAZ tip during early and late cell cycle stages, respectively (figures 7 and 8) [19], similar to the interdependence between CIF1 and TbPLK and between CIF1 and TbAUK1 (see above). Together, these results illustrate a regulatory scheme by which the CIF1-CIF2 complex recruits TbPLK to the new FAZ tip during S phase to G2 phase and, in turn, TbPLK maintains the CIF1-CIF2 complex at the new FAZ tip from G2 phase to early anaphase. During these cell cycle stages, TbPLK phosphorylates the CIF1-CIF2 complex, which may help the latter to recruit other downstream cytokinesis regulators, such as the KAT80-KAT60a complex, which depends on CIF1 to localize to the new FAZ tip at G2 phase [22], and TbAUK1, which depends on CIF1 and CIF2 to localize to the new FAZ tip at late anaphase [16,19]. During late anaphase when TbPLK disappears from the new FAZ tip, TbAUK1 appears to replace the role of TbPLK to maintain the CIF1-CIF2 complex at the new FAZ tip from late anaphase to cytokinesis and to phosphorylate the CIF1-CIF2 complex, which may enable the CIF1-CIF2 complex to recruit other downstream cytokinesis regulator(s), such as KLIF, which depends on CIF1 to localize to the new FAZ tip during late mitosis through interaction with CIF1 [22]. Altogether, these findings uncover a functional relay between TbPLK and TbAUK1 at the new FAZ tip during the cell cycle to cooperate with the CIF-CIF2 complex to promote cytokinesis initiation and, likely, cytokinesis completion.

The functional interplay among the cytokinesis regulatory proteins in *T. brucei* has been extensively investigated through the examination of the effect of knocking down of one regulator on the subcellular localization and protein stability of other regulators [16,19,20,22,25,34,40]. These investigations uncovered the interdependence between CIF1 and CIF2 for protein stability [19,34] and the interdependence between TbPLK and CIF3 [20,40], between KAT80 and CIF3 [22,40], between CIF3 and FPRC [40], between TbPLK and CIF1 (figure 4) [16], between TbPLK and CIF2 (figure 7) [19], between TbAUK1 and CIF1 (figure 5) [16] and between TbAUK1 and CIF2 (figure 8) [19] for the localization to the new FAZ tip. Additionally, other types of functional interplay between cytokinesis regulators were also observed. CIF1 and CIF4 are required for maintaining CIF3 stability, whereas CIF3 is necessary for the localization of CIF1 and CIF4 to the new FAZ tip [20,40]. Such complicated functional relationships among these cytokinesis regulators make it very difficult, if not impossible, to determine their order of actions in the cytokinesis pathway. However, our work on the functional relationships between CIF4 and CIF1 (electronic supplementary material, figure S8*a*,*b* and [25]), between CIF4 and FPRC (electronic supplementary material, figure S8*c*,*d*) [25], between CIF4 and TbPLK (figure 9*d*,*e*), and between FPRC and TbPLK (figure 9*f*,*g*) allowed us to place CIF4 upstream of FPRC, TbPLK and CIF1, and to place FPRC upstream of TbPLK and CIF1 in the cytokinesis pathway. TbAUK1 is known to be further downstream of TbPLK in the cytokinesis pathway, due to its emergence at the new FAZ tip during late mitosis [38] and its dependence on TbPLK and the CIF1-CIF2 complex for localization to the new FAZ tip [16,19]. Therefore, it is reasonable to speculate that TbAUK1 also functions downstream of CIF4 and FPRC in the cytokinesis pathway.

In summary, we identified CIF1 and CIF2 as *in vitro* substrates of TbPLK and TbAUK1 and mapped the phosphosites to the IDRs of CIF1 and CIF2. We also demonstrated that CIF1 and CIF2, which may function as a single unit, recruits TbPLK to the new FAZ tip during S phase and G2 phase, and subsequently TbPLK maintains CIF1 and CIF2 at the new FAZ tip from G2 phase to early anaphase. Further, we showed that at late anaphase the CIF1-CIF2 complex recruits TbAUK1 to the new FAZ tip, and subsequently TbAUK1 maintains CIF1 and CIF2 at the new FAZ tip after late anaphase. Finally, we showed that CIF4 and FPRC are not substrates of TbPLK and TbAUK1, and that they function in the upstream of the cytokinesis regulatory pathway by recruiting TbPLK to the new FAZ tip, likely through an indirect means via CIF1, which depends on CIF4 and FPRC to localize to the new FAZ tip and acts as a substrate to recruit TbPLK to the new FAZ tip. Altogether, these findings uncovered the functional interplay between the two conserved proteins kinases and those trypanosomatid-specific cytokinesis regulators and provided new insights into the mechanistic roles of these regulators in promoting cytokinesis in *T. brucei*.

## 4. Material and methods

### 4.1. Trypanosome cell culture

The *T. brucei* 29-13 strain [42] and Lister427 strain were cultured in SDM-79 medium supplemented with 10% heat-inactivated fetal bovine serum at 27°C. For the 29-13 strain, 15 µg ml^−1^ G418 and 50 µg ml^−1^ hygromycin were added into the culture medium. The following *T. brucei* cell lines have been reported previously; CIF1-3HA [16], TbAUK1-3HA [16], TbPLK-3HA [24], PTP-CIF2 [34], FPRC-PTP [25]. CIF1-3′UTR RNAi cell line expressing endogenous PTP-CIF1 [34], TbAUK1 RNAi cell line [16], TbPLK RNAi cell line [16], CIF1 RNAi cell line [16], CIF2 RNAi cell line [19], CIF4 RNAi cell line [25] and FPRC RNAi cell line [25]. These cell lines were cultured in SDM-79 medium containing appropriate antibiotics.

### 4.2. CIF1 RNAi complementation

The CIF1-3′UTR-RNAi cell line and the CIF1-3′UTR RNAi cell lines expressing triple HA-tagged full-length CIF1, CIF1-ΔCC, CIF1-ZnF1^mut^ and CIF1-ZnF2^mut^ have been previously reported [34]. To express the IDR-deletion mutants of CIF1 in the CIF1-3′UTR RNAi cell line expressing endogenously N-terminally PTP-tagged CIF1, DNA fragments for expressing CIF1-ΔIDR1 (deletion of a.a. 1–120) and CIF1-ΔIDR2 (deletion of a.a. 281–661) were cloned into pLew100-3HA-BLE vector, and the resulting plasmids were used to transfect the CIF1-3′UTR RNAi cell line. Successful transfectants were selected with 2.5 µg ml^−1^ phelomycin in addition to 1.0 µg ml^−1^ puromycin, 10 µg ml^−1^ blasticidin, 50 µg ml^−1^ hygromycin B and 15 µ ml^−1^ G418, and then cloned by limiting dilution in a 96-well plate containing SDM-79 medium supplemented with 20% fetal bovine serum and the above-mentioned antibiotics. To induce RNAi and ectopic expression of CIF1 and CIF1-deletion mutants, cells were incubated with 1.0 µg ml^−1^ tetracycline. Cell growth was monitored daily by counting the cell number under a light microscope, and cells were diluted with fresh medium every 3 days.

### 4.3. Endogenous epitope tagging of proteins

For tagging of CIF1, CIF2, FPRC, TbPLK and TbAUK1 from one of their endogenous loci, the one-step PCR-based epitope tagging method [43] was carried out. CIF1, CIF2 and FPRC were each tagged with an N-terminal PTP or triple Myc (for CIF2), whereas TbPLK and TbAUK1 were each tagged with a C-terminal triple HA epitope. PCR products were purified from agarose gel and used for transfection of *T. brucei* cells. Successful transfectants were selected with either 1.0 µg ml^−1^ puromycin, 10 µg ml^−1^ blasticidin or 40 µg ml^−1^ G418, depending on the plasmid constructs used for PCR and the *T. brucei* cell lines used for transfection, and transfectants were further cloned by limiting dilution in a 96-well plate containing SDM-79 medium supplemented with 20% fetal bovine serum and appropriate antibiotics.

### 4.4. Treatment of cells with GW843682X and Hesperadin and treatment of cell lysate with λPPase

GW843682X was originally developed as an inhibitor of human Plk1 [28], and it inhibits TbPLK activity, with an *in vitro* IC50 of approximately 1.3 µM and an *in vivo* IC50 of approximately 2 µM for the procyclic form of *T. brucei* [15]. Hesperidin was originally developed as an inhibitor of human Aurora B kinase [37], and it inhibits TbAUK1 activity, with an *in vitro* IC50 of approximately 40 nM and an *in vivo* IC50 of approximately 0.55 µM for the bloodstream form of *T. brucei* [36]. To inhibit TbPLK and TbAUK1 activity in *T. brucei* cells, cells were incubated with 5 µM GW843682X or 1 µM Hesperadin in SDM-79 medium at 27°C.

*Trypanosoma brucei* cells (5 × 10^6^) expressing CIF4-3HA was lysed in 100 µl of cell lysis buffer (25 mM Tris–HCl, pH 7.4, 100 mM NaCl, 1 mM DTT, 0.1% NP-40, and protease inhibitor mixture), and then 88 µl of cell lysate was incubated with 1.0 µl of *λ*PPase (40 units µl^−1^, Sigma-Aldrich), 1 µl of 10 mM MnCl_2_ and 10 µl of 10× *λ*PPase buffer (Sigma-Aldrich) at 30°C for 30 min. Phosphatase reaction was stopped by adding 1× SDS sampling buffer and boiled for 5 min before loading onto SDS-PAGE.

### 4.5. Purification of recombinant GST-fusion proteins and *in vitro* GST pull-down assays

The plasmids for expressing GST-KD^TbPLK^ (a.a. 1–314 of TbPLK), GST-PBD^TbPLK^ (a.a. 434–768 of TbPLK), GST-IDR1^CIF1^ (formerly GST-NTD^CIF1^, a.a. 1–120 of CIF1), GST-CC^CIF1^ (a.a. 121–271 of CIF1), GST-IDR2^CIF1^ (formerly GST-IDR^CIF1^, a.a. 272–666 of CIF1), GST-ZnF^CIF1^ (a.a. 667–804 of CIF1) and GST-CIF1 have been reported previously [22,25,44]. For expressing GST-CIF2 and GST-TbAUK1, the full-length coding sequences of CIF2 and TbAUK1 were each cloned into the pGEX-4T-3 vector (Clontech). These plasmids were each transformed into the *E. coli* BL21 strain. Expression of the GST-fused proteins was induced with 0.1 mM isopropyl β-d-thio-galactopyranoside (IPTG) for 4 h at room temperature or 15°C overnight, and recombinant proteins were purified through the glutathione sepharose beads. Purified recombinant GST-fusion proteins bound to the beads were then incubated with the lysate of *T. brucei* cells expressing triple HA-tagged CIF1, CIF1 IDR-deletion mutants, CIF1 ZnF point mutants, TbPLK, TbAUK1, FPRC or CIF4, or the lysate of *T. brucei* cells expressing PTP-CIF2. *Trypanosoma brucei* cell lysate was prepared by incubating the cells with the cell lysis buffer (see above) on ice for 30 min and cleared by centrifugation. Proteins bound to the glutathione sepharose beads were washed four times with the cell lysis buffer, eluted by boiling the beads in 1x SDS sampling buffer for 5 min, separated by SDS-PAGE, transferred onto a polyvinylidene difluoride (PVDF) membrane, and immunoblotted with anti-HA antibody to detect triple HA-tagged proteins or with anti-Protein A antibody to detect PTP-CIF2. GST alone was used as the negative control. GST and GST-fusion proteins used for pull-down were stained with Coomassie blue dye.

### 4.6. *In vitro* kinase assay using the thiophosphorylation method

*In vitro* kinase assay using thiophosphorylation was carried out according to the method developed previously, which uses a semisynthetic epitope for detection of thiophosphorylated kinase substrates [32]. *Trypanosoma brucei* cells expressing TbPLK-3HA, TbPLK-K70R-3HA, TbAUK1-3HA or TbAUK1-K58R-3HA were lysed in the cell lysis buffer (see above), and cell lysate was cleared by centrifugation in a microcentrifuge. Cleared cell lysate was incubated with the EZview Red anti-HA affinity gel (Sigma-Aldrich) for 30 min at 4°C, and the beads were washed three times with the cell lysis buffer and then two times with the kinase assay buffer (10 mM HEPES, pH7.6, 50 mM NaCl and 10 mM MgCl_2_). Subsequently, the beads were mixed with purified GST-IDR1^CIF1^, GST-IDR2^CIF1^ and GST-CIF2 in the kinase assay buffer plus 1 mM ATP-γ-S and incubated for 30 min at room temperature. Then, 50 mM alkylating agent *p*-nitrobenzylmesylate (PNBM) was added and incubated for 60 min at room temperature. The supernatant was then loaded onto SDS-PAGE, and proteins were transferred onto a PVDF membrane and blotted with the anti-ThioP monoclonal antibody, which recognizes the thiophosphate ester (1 : 5000 dilution, ThermoFisher). GST-fusion proteins were stained with Coomassie blue dye. The immunoprecipitated triple HA-tagged wild-type and kinase-dead mutant of TbPLK and TbAUK1 were eluted and separated by SDS-PAGE, transferred onto a PVDF membrane, and blotted with anti-HA antibody.

### 4.7. *In vitro* kinase assay and mass spectrometry

*Trypanosoma brucei* cells expressing TbPLK-3HA or TbAUK1-3HA were lysed, and cleared cell lysate was incubated with the EZview Red anti-HA affinity gel (Sigma-Aldrich) for 30 min at 4°C. Beads were then washed three times with the cell lysis buffer and two times with the kinase assay buffer (10 mM HEPES, pH 7.6, 50 mM NaCl, 10 mM MgCl_2_, 1 mM EGTA and 1 mM DTT). Subsequently, beads were incubated with purified GST-IDR1^CIF1^, GST-IDR2^CIF1^ or GST-CIF2 in the kinase assay buffer plus 0.2 mM ATP at room temperature for 30 min. Kinase reaction was stopped by adding 1× SDS-PAGE sample buffer to the reaction solution, and proteins were eluted by boiling for 5 min. Eluted proteins were separated by SDS-PAGE, and then stained with Coomassie blue dye. The gel slice containing each of the GST-fusion proteins was excised and analysed by LC-MS/MS.

Protein band in the excised gel slice was digested with trypsin according to published procedures [45]. Excised protein band was digested with 160 ng trypsin for 4 h at 37°C, and peptides were extracted with 50 ml of 50% acetonitrile and 5% formic acid. Extracted peptides were dried using SpeedVac, resuspended in 2% acetonitrile and 0.1% formic acid and injected onto Thermo LTQ Orbitrap XL (ThermoFisher Scientific), following published procedures [46]. Samples were analysed on an LTQ Orbitrap XL interfaced with an Eksigent nano-LC 2D plus ChipLC system (Eksigent Technologies). Samples were loaded onto a ChromXP C18-CL trap column (200 mm i.d. × 0.5 mm length) at a flow rate of 3 nl min^−1^. Reverse-phase C18 chromatographic separation of peptides was carried on a ChromXP C18-CL column (75 mm i.d × 10 cm length) at 300 nl min^−1^. The LTQ Orbitrap was operated in a data-dependent mode to simultaneously measure full-scan MS spectra in the Orbitrap and the five most intense ions in the LTQ by CID, respectively. In each cycle, MS1 was acquired at a target value of 1E6 with a resolution of 100 000 (*m/z* 400) followed by top five MS2 scan at a target value of 3E4. The mass spectrometric setting was as follows: spray voltage was 1.6 KV, charge state screening and rejection of singly charged ion were enabled. Ion selection thresholds were 8000 for MS2, 35% normalized collision energy, activation *Q* was 0.25, and dynamic exclusion was employed for 30 s. Raw data files were processed and searched against the *T. brucei* proteome database using the Mascot and Sequest HT (v. 13) search engines. The search conditions used were as follows: peptide tolerance of 10 p.p.m. and MS/MS tolerance of 0.8 Da, with two missed cleavages permitted and the enzyme set as trypsin.

### 4.8. Immunofluorescence microscopy

Cells were attached to the coverslips for 30 min at room temperature, fixed with cold methanol (−20°C) for 30 min, and rehydrated with PBS for 10 min. Cells on the coverslips were blocked with 3% BSA in PBS for 30 min at room temperature, and incubated with anti-CC2D polyclonal antibody (1 : 2000 dilution) [47], anti-CIF1 polyclonal antibody (1 : 1000 dilution) [22], anti-CIF2 polyclonal antibody (1 : 1000 dilution) [39], anti-Protein-A polyclonal antibody (Sigma-Aldrich, 1 : 400 dilution) or FITC-conjugated anti-HA antibody (Sigma-Aldrich, clone HA-7, 1 : 400 dilution) for 1 h at room temperature, or with anti-TbPLK polyclonal antibody (1 : 160 dilution) [17] for 1 h at 37°C. After the cells on the coverslips were washed three times with PBS, cells were incubated with Cy3-conjugated anti-rabbit IgG (Sigma-Aldrich, 1 : 400 dilution) for 1 h at room temperature. After three washes with PBS, the coverslips were mounted in DAPI-containing VectaShield mounting medium (Vector Laboratories) and examined using an inverted microscope (model IX71, Olympus) equipped with a cooled CCD camera (model Orca-ER, Hamamatsu). Images were acquired and processed with the Slidebook software (Intelligent Imaging Innovations).

## Data Availability

All data were included in the main figures and the accompanying electronic supplementary material, figures S1–S8 [48]. The raw mass spectrometry data have been deposited into the ProteomeXchange Consortium via the PeptideAtlas partner repository with the dataset identifier PASS02778 and password JX7325b.
